# Trends and Risk Factors for Antimicrobial-Resistant *Neisseria gonorrhoeae*, Melbourne, Australia, 2007 to 2018

**DOI:** 10.1128/AAC.01221-19

**Published:** 2019-09-23

**Authors:** Deborah A. Williamson, Christopher K. Fairley, Benjamin P. Howden, Marcus Y. Chen, Kerrie Stevens, Vesna De Petra, Ian Denham, Eric P. F. Chow

**Affiliations:** a Microbiological Diagnostic Unit Public Health Laboratory, Department of Microbiology and Immunology, The University of Melbourne at The Doherty Institute for Infection and Immunity, Melbourne, Australia; b Melbourne Sexual Health Centre, Alfred Health, Carlton, Australia; c Central Clinical School, Faculty of Medicine, Nursing and Health Sciences, Monash University, Melbourne, Australia

**Keywords:** *Neisseria gonorrhoeae*, antibiotic resistance, epidemiology, multidrug resistance, sexually transmitted diseases

## Abstract

Antimicrobial resistance (AMR) in Neisseria gonorrhoeae is a major public health problem. Traditionally, AMR surveillance programs for N. gonorrhoeae have focused mainly on laboratory data to describe the prevalence and trends of resistance. However, integrating individual-level risk factors (e.g., sexual orientation or international travel) with laboratory data provides important insights into factors promoting the spread of resistant N. gonorrhoeae. Here, over a 12-year period, we assessed the trends and risk factors for resistant N. gonorrhoeae in individuals attending a large publicly funded sexual health center in Melbourne, Australia. A total of 7,588 N. gonorrhoeae isolates were cultured from 5,593 individuals between 1 January 2007 and 31 December 2018. The proportion of isolates with penicillin resistance decreased from 49.5% in 2007 to 18.3% in 2018 (*p*_trend_ < 0.001) and from 63.5% in 2007 to 21.1% in 2018 for ciprofloxacin resistance (*p*_trend_ < 0.001). In contrast, the proportion of isolates displaying decreased susceptibility to ceftriaxone increased from 0.5% in 2007 to 2.9% in 2018 (*p*_trend_ < 0.001), with a significant increase in low-level azithromycin resistance, from 2.5% in 2012 to 8.2% in 2018 (*p*_trend_ < 0.001). Multivariate analysis identified risk factors for multidrug-resistant (MDR) N. gonorrhoeae, namely, female sex and country of birth, with MDR isolates more common in individuals born in northeast Asia, further highlighting the importance of this region and international travel as factors in the cross-border transmission of MDR N. gonorrhoeae. Future surveillance work should incorporate additional epidemiological and genomic data to provide a comprehensive overview of the emergence and spread of resistant N. gonorrhoeae.

## Abstract (precis)

Antimicrobial resistance (AMR) in Neisseria gonorrhoeae is a major public health problem. Traditionally, AMR surveillance programs for N. gonorrhoeae have focused mainly on laboratory data to describe the prevalence and trends of resistance. However, integrating individual-level risk factors (e.g., sexual orientation or international travel) with laboratory data provides important insights into factors promoting the spread of resistant N. gonorrhoeae.

## INTRODUCTION

Gonorrhea is a common sexually transmitted infection (STI) and represents a major public health problem globally. Untreated, gonorrhea can lead to severe sequelae, most notably pelvic inflammatory disease, ectopic pregnancy, and infertility in women (1). Moreover, gonorrhea infection can promote the transmission of other STIs, including HIV (2). A recent World Health Organization (WHO) report suggested there were 87 million new cases of gonorrhea in 2016 (3). In Australia, the incidence of gonorrhea has increased markedly over the past 5 years from 66.9 per 100,000 population in 2014 to 125.5 per 100,000 population in 2018 (4). Although the increase in gonorrhea incidence is likely to be partly accounted for by increases in sensitivity of laboratory testing with the introduction of nucleic acid amplification tests (NAATs) (5), increasing rates are likely to also reflect a rising disease burden, supported by a temporally concurrent increase in other bacterial STIs in Australia, such as chlamydia and syphilis (4, 6).

Compared to other sexually transmitted bacteria, Neisseria gonorrhoeae has an extraordinary capacity to develop and retain antimicrobial resistance (AMR) (1). The U.S. Centers for Disease Control and Prevention (CDC) classified N. gonorrhoeae as an “urgent resistance threat,” and it is also considered a “priority organism” in the Australian National Antimicrobial Resistance Strategy (7). At present in Australia, as in many other developed countries, dual therapy with ceftriaxone (500 mg intramuscularly plus azithromycin at 1 g orally) is recommended for the treatment of uncomplicated urogenital gonorrhea (8). However, in the context of reports of increasing low-level resistance to azithromycin and transmission of high-level azithromycin-resistant isolates, in January 2019 the British Association for Sexual Health and HIV (BASHH) national guideline for the management of gonorrhea has recommended ceftriaxone be used as monotherapy (increased from 500 mg to 1 g intramuscularly), with ciprofloxacin as an alternative agent if phenotypic or genotypic susceptibility testing data are available and indicate sensitivity to ciprofloxacin (9). Concerningly, however, N. gonorrhoeae strains with high-level ceftriaxone resistance have been increasingly reported (10) and, as the incidence of gonorrhea continues to increase, there will be further potential for the development and spread of resistant strains. Therefore, improved understanding of the sources and transmission of AMR in N. gonorrhoeae is urgently required.

Although many countries, including Australia, have national surveillance programs to monitor AMR in N. gonorrhoeae (11), few studies to date have combined AMR data with epidemiological and individual-level behavioral risk factors. Such information can provide important evidence-based insights into factors promoting the acquisition of resistant N. gonorrhoeae, such as sexual orientation, international travel, or sexual behaviors. Identification of specific risk groups may enable targeted public health action, such as intensified screening, contact tracing, or closer monitoring for failure of empirical therapy. In order to determine specific risk factors for acquiring AMR N. gonorrhoeae in our setting, we undertook a longitudinal observational study between 2007 and 2018 of all individuals with culture-confirmed gonorrhea presenting to a large urban sexual health center in Melbourne, Australia.

## RESULTS

### Baseline patient characteristics.

A total of 7,588 N. gonorrhoeae isolates were cultured from 5,593 individuals between 1 January 2007 and 31 December 2018. The number of isolates received for culture increased from 192 in 2007 to 1,511 in 2018 (see Table S2 in the supplemental material). Most individuals were male (5,132/5,593; 91.8%), and the median age of all individuals was 28 years (interquartile range [IQR], 24 to 34 years) (Table 1). Of the 442 female individuals, 133 (32%) reported current sex work. A total of 602/5,593 (10.8%) individuals were HIV positive, and 1,163/5,593 (20.8%) individuals had more than one episode of infection associated with a culture isolate during the study period (Table 1).

**TABLE 1:** Baseline characteristics of 5,593 individuals with culture-positive N. gonorrhoeae attending Melbourne Sexual Health Centre, January 2007 to December 2018^*a*^

Characteristic	No. of subjects (%)^*b*^			
	All individuals (*n* = 5,593)^*b*^	MSM (*n* = 4,470)	Heterosexual males (*n* = 662)	Females (*n* = 442)
Median age, yr [IQR]	28 [24–34]	28 [24–34]	30 [25–40]	27 [23–32]
				
Yr				
2007	184 (3)	146 (3)	26 (4)	11 (3)
2008	173 (3)	134 (3)	23 (3)	16 (4)
2009	240 (4)	178 (4)	42 (6)	20 (5)
2010	310 (6)	245 (5)	33 (5)	32 (8)
2011	337 (6)	257 (6)	53 (8)	27 (6)
2012	427 (8)	337 (8)	60 (9)	29 (7)
2013	537 (10)	434 (10)	79 (12)	20 (5)
2014	525 (9)	430 (10)	55 (8)	36 (9)
2015	590 (11)	501 (11)	53 (8)	34 (8)
2016	573 (10)	473 (11)	57 (9)	37 (9)
2017	786 (14)	636 (14)	78 (12)	66 (16)
2018	911 (16)	699 (16)	103 (16)	94 (22)
				
Region of birth				
Oceania	3,196 (57)	2,679 (60)	328 (50)	176 (42)
Northwest Europe	469 (8)	327 (7)	98 (15)	44 (10)
Southern and Eastern Europe	162 (3)	110 (2)	40 (6)	12 (3)
North Africa and Middle East	92 (2)	66 (1)	17 (3)	6 (1)
Southeast Asia	499 (9)	411 (9)	34 (5)	37 (9)
Northeast Asia	363 (6)	218 (5)	44 (7)	101 (24)
Southern and Central Asia	139 (2)	111 (2)	25 (4)	3 (1)
Americas	323 (6)	278 (6)	27 (4)	16 (4)
Sub-Saharan Africa	67 (1)	54 (1)	7 (1)	5 (1)
Unknown	283 (5)	216 (5)	42 (6)	22 (5)
				
Current sex worker				
Yes	205 (4)	52 (1)	8 (1)	133 (32)
No	4,718 (84)	3,877 (87)	601 (91)	228 (54)
Unknown	670 (12)	541 (12)	53 (8)	61 (14)
				
Sex with someone from overseas in preceding 12 mo				
Yes	1,482 (26)	1127 (25)	273 (41)	76 (18)
No	3,123 (56)	2,542 (57)	311 (47)	256 (61)
Unknown	988 (18)	801 (18)	78 (12)	90 (21)
				
Median no. [IQR] of male partners in preceding 12 mo	NA	6 [2–12]	NA	1 [0–4]
Median no. [IQR] of female partners in preceding 12 mo	NA	NA	3 [1–6]	NA
				
HIV status				
Positive	602 (11)	595 (13)	5 (1)	0 (0)
Negative	4,991 (89)	3,875 (87)	657 (99)	422 (100)

a Data are reported as the number of subjects (%) except as noted otherwise in column 1. The percentages represent the proportion of the total risk group. NA, not applicable; MSM, men who have sex with men; IQR, interquartile range.

b Nineteen individuals self-reported their gender as “other” or “transgender” and were not categorized into the three main risk groups.

### Trends in antimicrobial susceptibility.

**FIG 1:**
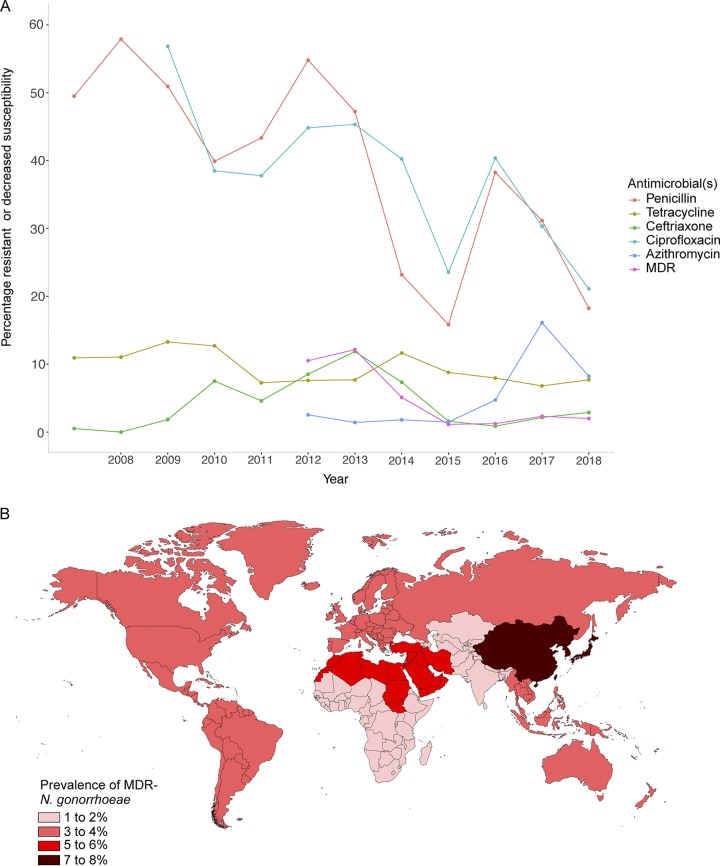
(A) Proportion of N. gonorrhoeae isolates from individuals attending Melbourne Sexual Health Centre displaying resistance or reduced susceptibility to antimicrobials, 2007 to 2018. (B) Geographic regions of birth and associated prevalence of MDR N. gonorrhoeae isolates in individuals included in this study.

The proportion of isolates with resistance to penicillin or ciprofloxacin decreased markedly during the study period, from 49.5% in 2007 to 18.3% in 2018 for penicillin (*p*_trend_ < 0.001) and from 63.5% in 2007 to 21.1% in 2018 for ciprofloxacin (*p*_trend_ < 0.001) (Fig. 1). The proportion of isolates displaying high-level tetracycline resistance remained relatively low and declined over the study period: 10.9% in 2007 to 7.7% in 2018 (*p*_trend_ < 0.001).

Overall, 4.1% (254/6,134) of isolates were classified as MDR between 2012 and 2018, and this level decreased significantly from 10.5% in 2012 to 2.0% in 2018 (*p*_trend_ < 0.001). However, when category 1 agents were considered separately, the proportion of isolates displaying reduced susceptibility to ceftriaxone increased significantly, from 0.5% in 2007 to 2.9% in 2018 (*p*_trend_ < 0.001), with a notable peak in 2013 at 11.9% (Fig. 1). There was a substantial increase in low-level azithromycin resistance from 2.5% in 2012 to 16.1% in 2017 (*p*_trend_ < 0.001), decreasing to 8.2% in 2018. Isolates from ten individuals displayed high-level resistance (HLR) to azithromycin (≥256 mg/liter): two in 2013, four in 2016, two in 2017, and two in 2018. Travel information was available for nine of these individuals; all had either had recent sexual contact overseas in Asia or had recent sexual contact with someone who had returned from Asia.

### Risk factors for AMR *N. gonorrhoeae*.

After adjustment for individual risk factors, the odds of having MDR N. gonorrhoeae decreased significantly over time (adjusted odds ratio [OR], 0.65; 95% confidence interval [95% CI], 0.61 to 070). In addition, the odds of having MDR N. gonorrhoeae among females was 1.96 times higher than in MSM (95% CI = 1.16 to 3.30). Figure 1 shows that the prevalence of MDR N. gonorrhoeae was highest (7.7%) among individuals born in Northeast Asia (covering China, Mongolia, Japan, and the Koreas), followed by individuals born in North Africa and the Middle East region (6.9%). Individuals born in Southern and Central Asia region had the lowest prevalence of MDR N. gonorrhoeae (1.8%). In the multivariable analysis, after adjustment for potential confounding factors, individuals born in Northeast Asia were more than twice as likely to have an MDR N. gonorrhoeae isolate compared to individuals born in Oceania (adjusted OR, 2.10; 95% CI = 1.33 to 3.32) (Table 2).

**TABLE 2:** Characteristics associated with MDR N. gonorrhoeae among 6,134 isolates from 4,612 individuals between 2012 and 2018^*a*^

Characteristics	MDR N. gonorrhoeae, *n/N* (%)	Initial OR and *P* value		Adjusted OR and *P* value	
		OR (95% CI)	*P*	OR (95% CI)	*P*
Yr		0.67 (0.63–0.72)	<0.001	0.65 (0.61–0.70)	<0.001
					
Age (yr)					
≤24	89/1,658 (5.4)	1.12 (0.73–1.73)	0.599		
25–34	108/2,947 (3.7)	0.75 (0.49–1.14)	0.182		
35–44	28/926 (3.0)	0.62 (0.36–1.05)	0.076		
≥45	29/601 (4.8)	1 (ref)			
					
Risk population					
MSM	204/5,230 (4.6)	1 (ref)		1 (ref)	
Heterosexual males	24/527 (3.9)	1.17 (0.76–1.81)	0.468	1.03 (0.66–1.60)	0.913
Females	25/334 (7.5)	1.99 (1.29–3.07)	0.002	1.95 (1.16–3.30)	0.012
Others^*b*^	1/43 (2.3)	0.59 (0.08–4.29)	0.602	0.78 (0.10–5.90)	0.806
					
Current sex work					
No	217/5,026 (4.3)	1 (ref)		1 (ref)	
Yes	12/157 (7.6)	1.84 (1.00–3.36)	0.049	0.95 (0.45–2.00)	0.901
Unknown	25/951 (2.6)	0.60 (0.40–0.91)	0.017	1.45 (0.92–2.28)	0.111
					
Sex overseas in preceding 12 mo					
No	137/3,201 (4.3)	1 (ref)			
Yes	74/1,525 (4.9)	1.14 (0.85–1.52)	0.375		
Unknown	43/1,408 (3.1)	0.71 (0.50–1.00)	0.052		
					
HIV status					
Negative	219/5,291 (4.1)	1 (ref)			
Positive	35/843 (4.2)	0.99 (0.69–1.44)	0.975		
					
Country of birth					
Oceania	151/3,628 (4.2)	1 (ref)		1 (ref)	
Northwest Europe	19/455 (4.2)	1.00 (0.61–1.63)	0.999	1.01 (0.61–1.67)	0.962
Southern and Eastern Europe	7/171 (4.1)	0.99 (0.46–2.15)	0.982	1.09 (0.50–2.40)	0.825
North Africa/Middle East	6/87 (6.9)	1.71 (0.73–3.99)	0.214	2.05 (0.86–4.88)	0.107
Southeast Asia	18/559 (3.2)	0.77 (0.46–1.26)	0.295	1.01 (0.61–1.68)	0.969
Northeast Asia	28/362 (7.7)	1.92 (1.26–2.93)	0.002	2.10 (1.33–3.32)	0.001
Southern and Central Asia	3/163 (1.8)	0.43 (0.14–1.38)	0.157	0.46 (0.14–1.47)	0.187
Americas	14/364 (3.8)	0.93 (0.53–1.62)	0.786	1.20 (0.68–2.12)	0.534
Sub-Saharan Africa	2/88 (2.3)	0.55 (0.14–2.28)	0.414	0.64 (0.15–2.64)	0.533
Unknown	6/257 (2.3)	0.55 (0.24–1.26)	0.159	0.49 (0.21–1.12)	0.091

a MDR, multidrug resistant; OR, odds ratio; ref, reference; *n/N*, number MDR/number in risk group.

b The category “Others” includes individuals who self-identified their gender as either “other” or “transgender.”

In addition to MDR N. gonorrhoeae, we also assessed changes over time in resistance to specific antimicrobials by sexual risk group. Among MSM, females, and heterosexual men, changes were generally similar, except that azithromycin resistance only rose significantly in MSM, reached a peak of 18% in 2017 and then dropped to 9% in 2018 (see Fig. S1 in the supplemental material).

## DISCUSSION

In this study, we integrated individual-level risk data with antimicrobial susceptibility results to determine risk factors for AMR N. gonorrhoeae in individuals presenting to a major publicly funded sexual health center in Australia over the last decade. Our data demonstrate considerable temporal variation in the prevalence of gonococcal resistance, with marked differences across key antimicrobials (particularly azithromycin, penicillin, and ciprofloxacin) and a substantial decline in the prevalence of MDR N. gonorrhoeae between 2012 and 2018. Our data also highlight the potential importance of international travel in the cross-border transmission of N. gonorrhoeae resistance from Asia to other parts of the globe.

Of note was the overall increase in low-level azithromycin resistance across the study period, a similar trend that has been observed in other countries, such as the United Kingdom and the United States (12, 13). Indeed, in light of increasing azithromycin resistance, the United Kingdom removed azithromycin as a first-line agent for the treatment of uncomplicated anogenital and oropharyngeal gonorrhea in January 2019 (9). In our study, the prevalence of azithromycin resistance peaked at 16.1% in 2017 but then decreased to 8.2% in 2018; however, in both years the prevalence of azithromycin resistance was above the WHO’s suggested 5% resistance threshold for changing treatment recommendations (14). Of note, the greatest increase in azithromycin resistance was in MSM, who are likely to have had the greatest exposure to azithromycin. Importantly, work in our setting has demonstrated an alarmingly high level of azithromycin resistance in other STIs and not just N. gonorrhoeae infections. For example, we recently identified an azithromycin resistance rate of over 90% among MSM-associated *Shigella* spp. in Victoria. (15). Similarly, macrolide resistance in M. genitalium exceeds 60% in Melbourne (16), with previous work demonstrating the potential for azithromycin exposure to be associated with macrolide-resistant isolates posttreatment (17). Although it is possible that increasing the dose of azithromycin from 1 to 2 g (as recently recommended in Australian guidelines for oropharyngeal gonorrhea) (8) may overcome the potential for gonorrhea treatment failures caused by low-level-resistant azithromycin isolates (18), it remains to be seen what further “collateral damage” may result from increasing azithromycin exposure, both at the patient and population levels. Of note, and unlike low-level azithromycin resistance, the majority of HLR azithromycin isolates in our study were from people with recent sexual contact (direct and indirect) in Asia, further highlighting this region as a potential reservoir for MDR-N. gonorrhoeae, as recently described (19).

The overall decline in resistance to penicillin and ciprofloxacin in our setting since 2012 is consistent with an observed national trend of decreased resistance to these agents in Australia (11), although the prevalence of resistance to both still remained relatively high in 2018 at 18.3 and 21.1%, respectively. One likely explanation for the decline in ciprofloxacin resistance is the widespread removal in the early to mid-2000s of ciprofloxacin as a recommended agent for the syndromic treatment of STIs (1), with a resultant decrease in population-level selection pressure. This situation has parallels with the decline in cefixime resistance in the United Kingdom, following the removal of cefixime as a recommended first-line agent (12). Of note, the 2019 UK guidelines also suggested that ciprofloxacin could again be used as a first-line agent, if either phenotypic or genotypic susceptibility is demonstrated (9). Recently, several PCR-based molecular assays for the direct detection of ciprofloxacin resistance in N. gonorrhoeae have been developed, including a commercially available assay (20, 21). These assays offer considerable promise in enhancing AMR surveillance in the absence of bacterial culture, i.e., when clinical specimens only undergo NAAT and could (depending on whether clinically relevant turnaround times can be achieved) be used to guide individualized treatment regimens based on their resistance profile. The approach of resistance-guided therapy has been applied in other STIs, particularly M. genitalium (16), but it has not yet been widely used for N. gonorrhoeae.

In our study, individuals born in Northeast Asia region had the highest prevalence of resistance in N. gonorrhoeae (19). Several factors may have contributed to this observation, for example, frequent travel to and from this region may increase the likelihood of resistant N. gonorrhoeae isolates being imported from overseas, similar to recent incursions of ceftriaxone- and/or azithromycin-resistant isolates into Australia (10). Furthermore, we found that females were more likely to have MDR N. gonorrhoeae in the adjusted analysis. Although previous studies have shown that sex work is associated with MDR N. gonorrhoeae (22, 23), this association was not observed in our study. It is possible that other unexplored factors, such as recent use of antibiotics and a history of STIs, may have contributed to MDR N. gonorrhoeae (24). In addition, it is possible that assortative sexual mixing between individuals of similar ethnicities may facilitate transmission of resistant isolates within distinct sexual networks, as suggested elsewhere (24). The limited association of MDR N. gonorrhoeae with other behavioral risk factors is similar to a recent study from the United Kingdom (25) and further highlights the complexity of understanding the drivers of the emergence and spread of AMR in N. gonorrhoeae, including the possible role of “bystander selection” (selection for resistance through exposure to antimicrobials used for a purpose other than STI treatment) in contributing to resistance (26).

There were a number of limitations to our study. First, our data are from a single site and may not be representative of gonococcal infections in other parts of Australia. Further, our data represent only cultured N. gonorrhoeae isolates and therefore represent only a proportion of all gonorrhea notifications. However, this limitation is not unique to our study and applies to most current N. gonorrhoeae AMR surveillance programs. Although ongoing culture-based N. gonorrhoeae AMR surveillance is essential, future surveillance programs should also attempt to incorporate information from clinical samples using existing (PCR-based) and emerging (e.g., metagenomic) molecular approaches. Further, whole-genome sequencing of isolates would provide additional insights into the emergence, transmission, and molecular determinants of resistance in N. gonorrhoeae, as described in several recent studies (27–29). In the context of increasingly resistant N. gonorrhoeae, it is critical that surveillance programs extend beyond simply reporting numbers of gonorrhea notifications and take advantage of all available sources of data, tools, and technologies.

## MATERIALS AND METHODS

### Setting, data sources, and definitions.

The Melbourne Sexual Health Centre (MSHC) is the major publicly funded sexual health service in Victoria, Australia, providing around 50,000 clinical consultations annually. No referrals are required, and all services are free of charge. Approximately 40% of the consultations are with men who have sex with men (MSM) (30). All clients who attend MSHC are required to complete a questionnaire using computer-assisted self-interview (CASI). Information recorded in the CASI includes basic demographic data, self-reported sexual practices, and recent sexual contact with someone from overseas. This information is linked into the MSHC electronic medical system, the Clinic Practice Management System (CPMS), at each clinic visit. The following information was extracted from the CPMS for all individuals who had a positive culture for N. gonorrhoeae at MSHC between 1 January 2007 and 31 December 2018: age, sex, sexual orientation (MSM, male heterosexuals, and all women), overseas sexual contact within the preceding 12 months (defined as having sex while traveling overseas or having sex in Australia with someone normally resident overseas), previous gonorrhea infection, geographic region of birth (categorized using the *Standard Australian Classification of Countries*, 2nd edition) (31), and HIV status.

### Antimicrobial susceptibility testing.

All N. gonorrhoeae isolates from MSHC between 1 January 2007 and 31 December 2018 underwent identification and antimicrobial susceptibility testing (AST) at the Microbiological Diagnostic Unit Public Health Laboratory (MDU PHL) in Victoria, Australia. Briefly, isolates were confirmed as N. gonorrhoeae based on Gram stain, oxidase, superoxol, and carbohydrate utilization tests or Vitek MS (matrix-assisted laser desorption ionization--time of flight; bioMérieux).

AST was performed in accordance with the Australian Gonococcal Surveillance Program (AGSP) using agar dilution for the following antimicrobials: penicillin (resistant, ≥1 mg/liter), ceftriaxone (decreased susceptibility, ≥0.06 mg/liter), ciprofloxacin (resistant, ≥1 mg/liter), spectinomycin (≥128 mg/liter), high-level tetracycline resistance (≥16 mg/liter), and—from January 2012 onward—azithromycin (resistant, ≥1 mg/liter) (32) (see Table S1 in the supplemental material). WHO strains F, G, K, N, and P were used as reference strains for AST, and quality assurance was performed through the National Neisseria Network (11).

Similar to a recent study (25), we modified the previously used definition of MDR N. gonorrhoeae by Tapsall et al. (33). We defined MDR N. gonorrhoeae as an isolate displaying resistance or decreased susceptibility to one or more first-line antimicrobials used to treat gonorrhea (category 1) and resistance to two or more antimicrobials less commonly used for treatment (category 2) (see Table S1 in the supplemental material). We restricted our analysis of MDR-N. gonorrhoeae from 2012 onward to reflect the widespread introduction of azithromycin into clinical treatment guidelines (8).

Where a patient had N. gonorrhoeae isolated from multiple anatomical sites at the same clinic visit, only one isolate was included in subsequent analyses. In males with multiple isolates, the isolates were included in the following order of preference: oropharyngeal, anorectal, and urethral. For females, the order of preference was oropharyngeal, genital, and then anorectal. This order of preference was chosen to facilitate comparison with data obtained from other gonococcal surveillance programs (34).

### Statistical analysis.

A χ^2^ test for trend was used to assess the changes in the proportion of isolates displaying resistance or reduced susceptibility for each antimicrobial and MDR over time. Univariable and multivariable generalized estimating equation (GEE) logistic regression models for repeated measures was used to identify patient-level factors associated with MDR N. gonorrhoeae between 2012 and 2018 (susceptibility testing for azithromycin was performed from 2012 onward). Variables with a *P* value of <0.10 in the univariable analyses were considered to be potential confounding factors and were included in the multivariable analysis. Analyses were performed using Stata (v14; StataCorp, College Station, TX).

### Ethics.

This study was approved by the Alfred Hospital Ethics Committee, Melbourne, Australia (study 545/17).

## Supplementary Material

Supplemental file 1
