# Supplementary material for: Trends and Risk Factors for Antimicrobial-Resistant Neisseria gonorrhoeae, Melbourne, Australia, 2007 to 2018
Source: Antimicrob Agents Chemother. 2019 Sep 23;63(10):e01221-19. doi: 10.1128/AAC.01221-19 (PMC6761556; doi:10.1128/AAC.01221-19)
Supplement: Supplemental file 1 [file AAC.01221-19-s0001.pdf]

SUPPLEMENTARY MATERIAL

Table S1: Australian Gonococcal Surveillance Program (AGSP) interpretative criteria for *Neisseria gonorrhoeae* Minimum Inhibitory Concentration (MIC) values.

**Antimicrobial**	**MIC (mg/L)**		
	**Sensitive**	**Less susceptible / decreased susceptibility**	**Resistant**
**Category 1**			
Ceftriaxone	≤ 0.03	0.06–0.125	Not defined
Azithromycin	≤ 0.5	Not defined	≥1 [a]
**Category 2**			
Penicillin [b]	≤ 0.03	0.06–0.5	≥1
Ciprofloxacin	≤ 0.03	0.06–0.5	≥1
Spectinomycin	≤64	Not defined	≥128
Tetracycline	≤ 8	Not defined	≥16 [c]

[a] Low-level resistance defined as 1-128 mg/L; high-level resistance defined as ≥256 mg/L (1).

[b] All penicillinase-producing *N. gonorrhoeae* (PPNG) isolates are considered resistant to penicillin, irrespective of the MIC value.

[c] The AGSP monitors for high level resistance to tetracycline (TRNG) only. Tetracyclines are rarely, if ever used for treatment of gonorrhoea in Australia. As such, TRNG is used for epidemiological surveillance purposes only.

Table S2: Number of *Neisseria gonorrhoeae* isolates received for antimicrobial surveillance testing from individuals attending Melbourne Sexual Health Centre, January 2007 – December 2018.

Year	Number of isolates
2007	192
2008	191
2009	271
2010	387
2011	413
2012	551
2013	708
2014	723
2015	878
2016	805
2017	1,117
2018	1,352

REFERENCES

1. Lahra MM, Enriquez RP. 2017. Australian Gonococcal Surveillance Programme annual report, 2015. Commun Dis Intell Q Rep 41:E.

Supplementary Figure 1. Proportion of *Neisseria gonorrhoeae* isolates from individuals attending Melbourne Sexual Health Centre displaying resistance or reduced susceptibility to antimicrobials, 2007 – 2018 stratified by sexual risk group: (A) Heterosexual males; (B) Men who have sex with men (MSM); (C) Females.

A

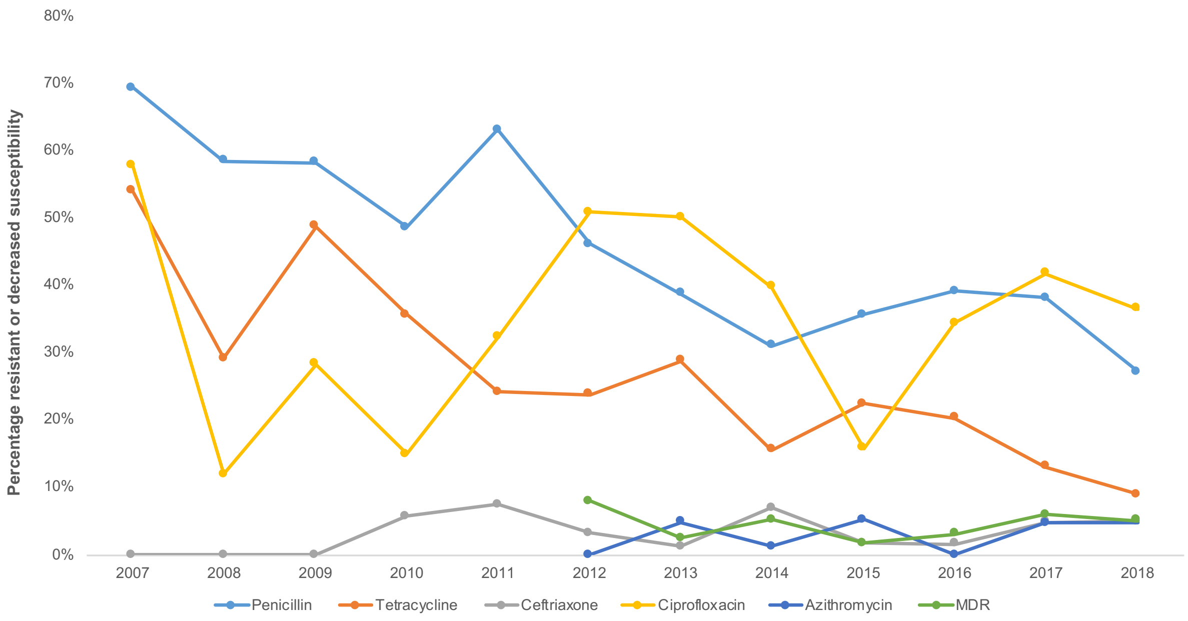

B

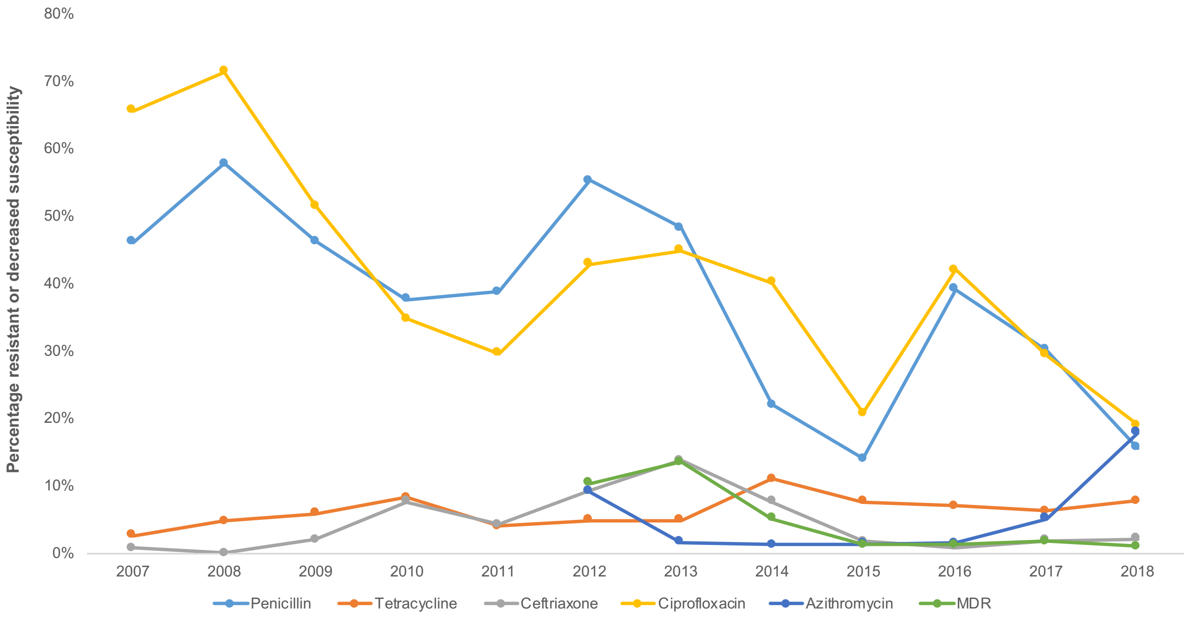

C

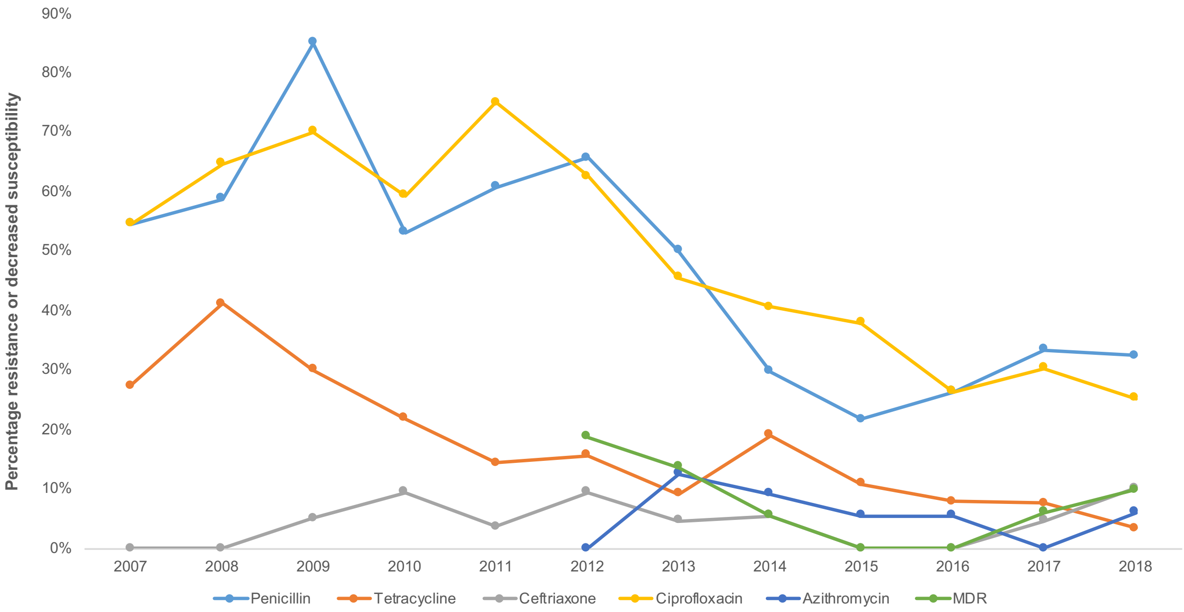

Percentage resistance or decreased susceptibility
90%
80%
70%
60%
50%
40%
30%
20%
10%
0%
2007
2008
2009
2010
2011
2012
2013
2014
2015
2016
2017
2018
Penicillin
Tetracycline
Ceftriaxone
Ciprofloxacin
Azithromycin
MDR
